# 822 12-Year Institutional Experience with Pediatric Lower Extremity Burns

**DOI:** 10.1093/jbcr/iraf019.353

**Published:** 2025-04-01

**Authors:** Hilary Liu, Christopher Fedor, Mare Kaulakis, Alexis Henderson, José Arellano, Garth Elias, Alain Corcos, Jenny Ziembicki, Francesco Egro

**Affiliations:** University of Pittsburgh Medical Center; University of Pittsburgh School of Medicine; University of Pittsburgh School of Medicine; University of Pittsburgh School of Medicine; University of Pittsburgh Medical Center; University of Pittsburgh Medical Center; University of Pittsburgh Medical Center; University of Pittsburgh Medical Center; University of Pittsburgh Medical Center

## Abstract

**Introduction:**

Lower extremity burns can lead to significant morbidity and long-term functional impairment, especially in pediatric patients, whose management and outcomes are not well understood. This study explores the management and outcomes of pediatric lower extremity burns.

**Methods:**

A retrospective cohort study was conducted on patients under the age of 18 years who presented to a single ABA-verified burn center from 2012 to 2023 with lower extremity burns. Data collected included demographics, burn characteristics, treatment strategies, and outcomes.

**Results:**

The study included 121 patients (52.9% male, 47.1% female; mean age 5.6±5.1 years). The most common burn etiologies were scald (n=62; 51.7%), flame (n=34; 28.3%), and contact (n=16; 13.3%), with one (0.8%) electrical and two (1.7%) chemical burns. The mean total body surface area (TBSA) affected was 8.6±11.7%, with a lower extremity BSA of 4.2±4.7%. Most burns were superficial partial-thickness (n=51; 43.2%), deep partial-thickness (n=37; 31.4%), or full-thickness (n=24; 20.3%). Commonly burned areas included the thigh (n=68; 56.2%), foot (n=50; 41.3%), and lower leg (n=45; 37.2%). Five patients (4.1%) had burns to the entire lower extremity.

Most patients (n=82; 68.3%) received conservative treatment, primarily antimicrobial cream or ointment (n=56; 68.3%), silver dressing (n=47; 57.3%), and chemical debridement agents (n=28; 34.1%). Surgical intervention was needed for 38 patients (31.7%), with common procedures being burn wound excision and autografting (n=19; 50.0%) and a two-stage operation with cadaver graft (n=13; 34.2%). The mean time from injury to the first operation was 5.1±5.1 days, with an average of 1.3±1.3 operations until closure. Ten patients (26.3%) required re-intervention for complications, including one (2.6%) with partial graft loss, one total graft loss, and eight (21.1%) with soft tissue infections.

**Conclusions:**

Despite the prevalence of deep burns in pediatric patients, surgical intervention remains limited, with many receiving conservative treatment. Effective infection control and treatment of hypertrophic scarring are crucial considerations in this population to optimize long-term outcomes and functional recovery.

**Applicability of Research to Practice:**

This study emphasizes the need for tailored management strategies in pediatric lower extremity burns, particularly focusing on effective infection control and hypertrophic scarring prevention to improve patient outcomes.

**Funding for the Study:**

N/A

